# Supplementary figures and images for: Improved isolation and detection of toxigenic Vibrio parahaemolyticus from coastal water in Saudi Arabia using immunomagnetic enrichment
Source: PeerJ. 2021 Oct 29;9:e12402. doi: 10.7717/peerj.12402 (PMC8559605; doi:10.7717/peerj.12402)

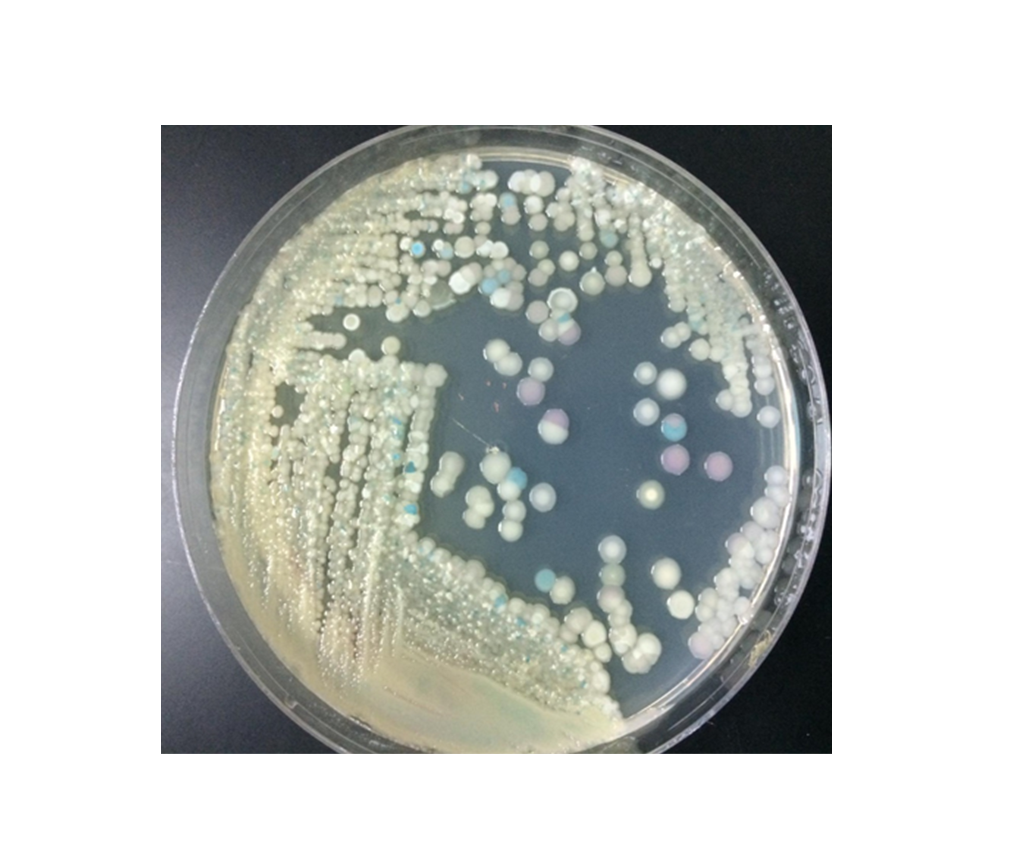

Supplement: Supplemental Information 1 [file peerj-09-12402-s001.png]

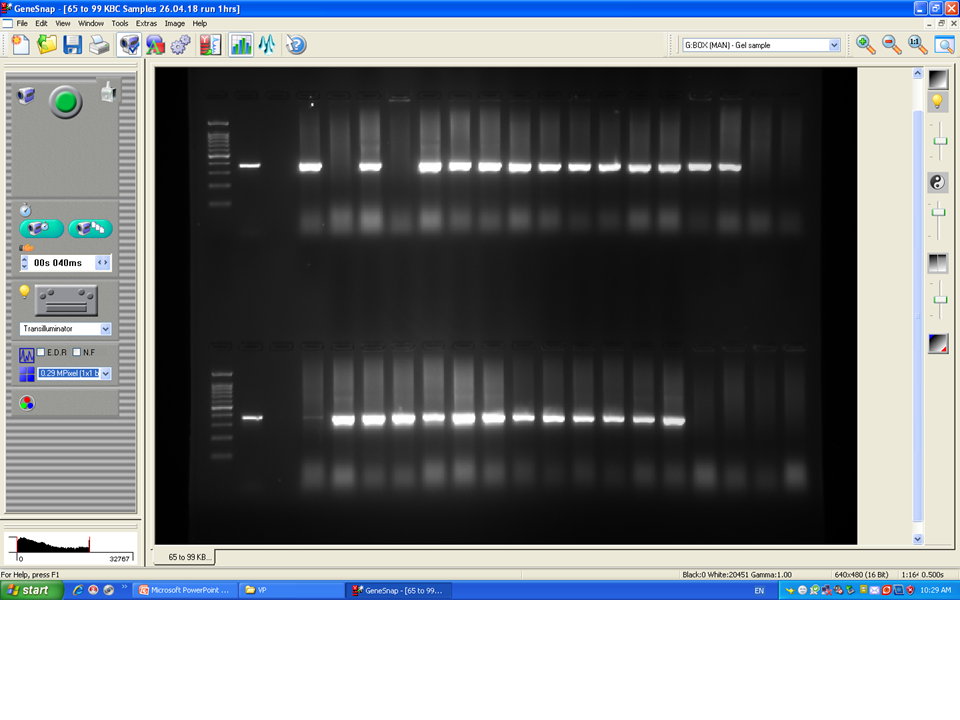

Supplement: Supplemental Information 2 [file peerj-09-12402-s002.png]

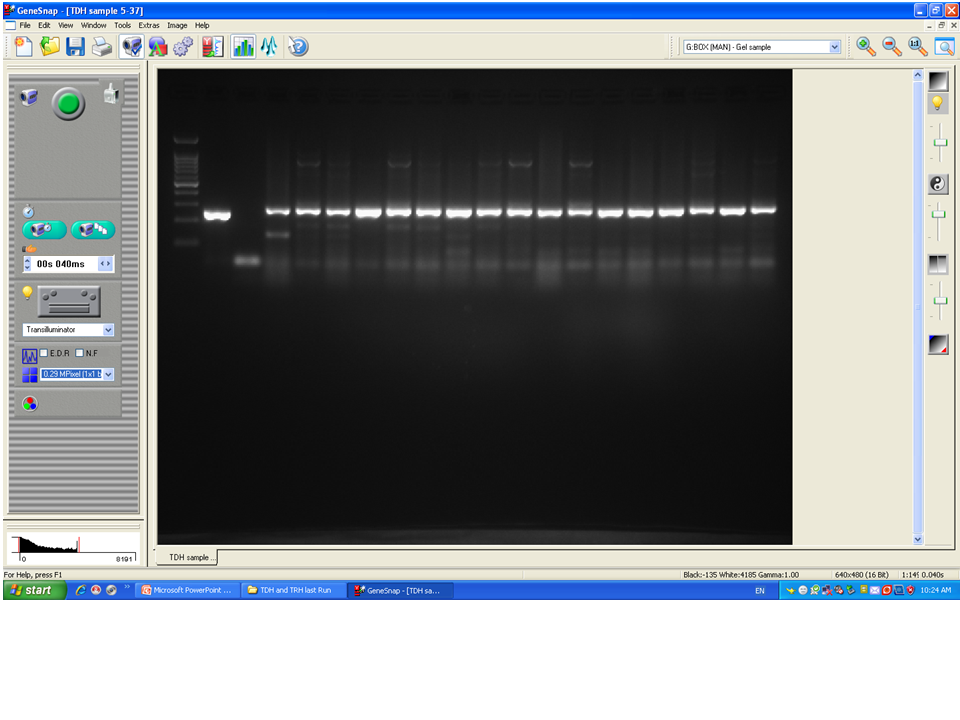

Supplement: Supplemental Information 3 [file peerj-09-12402-s003.png]

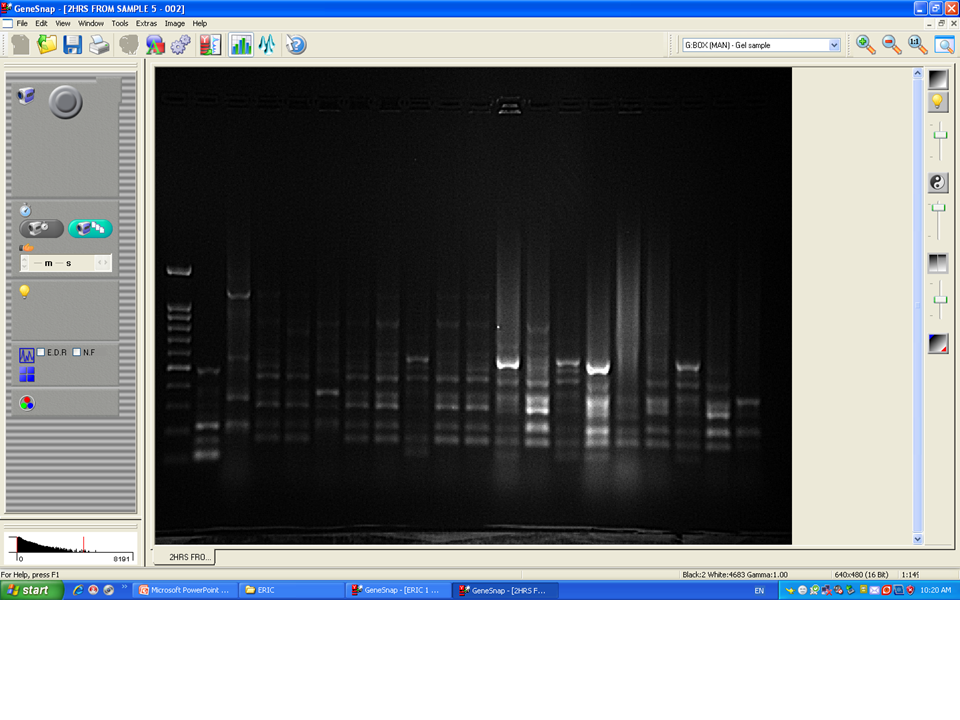

Supplement: Supplemental Information 4 [file peerj-09-12402-s004.png]
